# Temperature stress promotes cell division arrest in *Xanthomonas citri* subsp. *citri*

**DOI:** 10.1002/mbo3.323

**Published:** 2015-12-13

**Authors:** Júlia A. P. Sumares, Luana Galvão Morão, Paula M. M. Martins, Daniela A. B. Martins, Eleni Gomes, José Belasque, Henrique Ferreira

**Affiliations:** ^1^ Faculdade de Ciências FarmacêuticasDepto. de Ciências BiológicasUniversidade Estadual PaulistaRodovia Araraquara/Jaú Km 1, CP 502AraraquaraSP14801‐902Brazil; ^2^ Depto. Bioquímica e MicrobiologiaInstituto de BiociênciasUniversidade Estadual PaulistaAv. 24A 1515Rio ClaroSP13506‐900Brazil; ^3^ Depto. de Bioquímica e Tecnologia QuímicaInstituto de QuímicaUniversidade Estadual PaulistaR. Prof. Francisco Degni, 55AraraquaraSP14800‐060Brazil; ^4^ Depto. de BiologiaUniversidade Estadual PaulistaRua Cristóvão Colombo2265 Jardim NazarethSão Jose do Rio PretoSP15054‐000Brazil; ^5^ Depto. de Fitopatologia e NematologiaEscola Superior de Agricultura “Luiz de Queiroz”Universidade de São PauloAv. Pádua Dias 11PiracicabaSP13418‐900Brazil

**Keywords:** Cell division, chromosome segregation, citrus canker, thermal stress

## Abstract

Citrus canker is an economically important disease that affects orange production in some of the most important producing areas around the world. It represents a great threat to the Brazilian and North American citriculture, particularly to the states of São Paulo and Florida, which together correspond to the biggest orange juice producers in the world. The etiological agent of this disease is the Gram‐negative bacterium *Xanthomonas citri* subsp. *citri* (Xcc)*,* which grows optimally in laboratory cultures at ~30°C. To investigate how temperatures differing from 30°C influence the development of Xcc, we subjected the bacterium to thermal stresses, and afterward scored its recovery capability. In addition, we analyzed cell morphology and some markers of essential cellular processes that could indicate the extent of the heat‐induced damage. We found that the exposure of Xcc to 37°C for a period of 6 h led to a cell cycle arrest at the division stage. Thermal stress might have also interfered with the DNA replication and/or the chromosome segregation apparatuses, since cells displayed an increased number of sister origins side‐by‐side within rods. Additionally, Xcc treated at 37°C was still able to induce citrus canker symptoms, showing that thermal stress did not affect the ability of Xcc to colonize the host citrus. At 40–42°C, Xcc lost viability and became unable to induce disease symptoms in citrus. Our results provide evidence about essential cellular mechanisms perturbed by temperature, and can be potentially explored as a new method for *Xanthomonas citri* synchronization in cell cycle studies, as well as for the sanitation of plant material.

## Introduction

Bacterial cell cycle synchronization is useful in the isolation of subgroups of cells within a culture exhibiting different morphologies and/or physiological behaviors. Among the techniques employed to obtain synchronization, centrifugation gradients have been widely explored in *Caulobacter crescentus* (Schrader and Shapiro 2015), a bacterial model for cell cycle and differentiation studies. This is probably the less disturbing method used since it does not require any specific cell lineage or mutant to be applied, and it allows the isolation of large amounts of cells (Lin et al. 2012; Schrader and Shapiro 2015). Synchronization can also be obtained by immobilizing cells onto solid surfaces followed by the rescue of the newborn cells that emerge after division (Helmstetter et al. 1992). While this method has been used to isolate newborn *Escherichia coli* (*E. coli*) cells, there are, however, concerns about its low yield, and the need for specific strains for it to be successful. More recently, cell cycle synchronization was obtained in *E. coli* by inducing the stringent response phenotype (Ferullo and Lovett 2008; Ferullo et al. 2009). This can be achieved by the inhibition of tRNA charging, using serine hydroxamate or overexpressing RelA, which leads to a DNA replication initiation arrest. Inhibition at the initiation of DNA replication was also reported in *E. coli* mutants carrying thermo‐sensitive *dnaC* alleles (Withers and Bernander 1998). Although this method of cell cycle arrest induced by thermal stress seems an attractive and easy procedure for cell synchronization, it has some disadvantages, such as the requirement for specific mutations, the occurrence of abnormal restarts of DNA replication following the stress, and the fact that the synchronization status cannot be kept for long periods.

*Xanthomonas citri* subsp. *citri* (syn. *Xanthomonas axonopodis* pv. citri; (Schaad et al. 2006, 2005)) is the etiological agent of citrus canker, which is one of the most important diseases affecting citrus crops worldwide. In the field, Xcc is exposed to thermal stress, especially at the epiphytic stage when the bacterium occupies the surfaces of citrus leaves. Xcc has a limited life outside the host tissues, and it spreads from plant to plant by a combination of wind and rain, without the requirement of an insect vector (Gottwald et al. 2002a; Graham et al. 2004). Epiphytic survival is apparently dependent on the ability of Xcc to produce the extracellular polysaccharide xanthan gum, which in turn has been implicated with the bacterial capability to form structured biofilms (Dunger et al. 2007; Rigano et al. 2007). Together, xanthan gum and biofilm may protect the cells against dehydration and contribute to a better/more efficient leaf surface attachment, therefore resulting in an improvement of host colonization.

To understand the effects that thermal stress has on growth (cell division/ proliferation) of Xcc, we exposed the bacterium to different temperatures for periods of 6 h. We observed that cells grown at 37°C can be synchronized in culture, an effect apparently induced by a detectable cell division arrest. By raising the temperature to the window of 40–42°C, we were able to define the heat limit that compromise cell viability and its ability to induce disease symptoms in citrus. Our data support the use of thermal stress as an attractive method to synchronize Xcc cells for various biological investigations, and a method that can contribute to the development of sanitation protocols to eliminate Xcc from plant material.

## Materials and Methods

### Bacterial strains and media

The wild‐type Xcc strain used was the isolate formerly designated as *Xanthomonas axonopodis* pv. citri strain 306 (IBSBF‐1594), sequenced by (da Silva et al. 2002). Xcc *amy*::pPM2a‐*zapA* is a mutant of Xcc labeled for the divisional septum (Martins et al. 2010). Xcc *parB*::pAPU3, expressing ParB‐GFP that labels the segregating chromosome replication origins, was described previously (Ucci et al. 2014). Bacteria were cultivated in NYG/NYG‐agar medium (Peptone 5 g/L, yeast extract 3 g/L and glycerol 20 g/L) at 30°C (unless otherwise stated), which is considered the optimum temperature for Xcc growth. Kanamycin was added to the media at 20 *μ*g/mL when cultivating the mutants Xcc *amy*::pPM2a‐*zapA* and Xcc *parB*::pAPU3.

### Growth curves

A permanent culture of wild‐type Xcc was activated in NYG‐agar plate for 48 h at 30°C. After growth, isolated colonies were restreaked on the same medium and incubated for further 48 h in order to produce biomass. A bacterial suspension to be used as inoculum was prepared by dissolving Xcc biomass from the second growth in 50 mL of NYG‐medium using a polypropylene tube. The OD_600 nm_ of the cell suspension was determined using a Spectrophotometer DU‐730 (Beckman Coulter), and cells were used to inoculate six 125 mL Erlenmeyer flasks containing 50 mL NYG‐medium so to give a starting OD_600 nm_ of ~0.01. Cultures were initially incubated at 30°C and agitated at 200 rpm in a refrigerated rotary shaker (Innova 4230; New Brunswick Scientific, Edison, NJ). When cultures reached the OD_600 nm_ of ~0.4, which pilot experiments indicated to be the beginning of the exponential phase (generally after 12 h of growth), three flasks were kept at 30°C to serve as a control; the remaining three flasks were then transferred to another refrigerated shaker (ProBlot 12S; Labnet, Edison, NJ.), previously equilibrated at one of the testing temperatures (20, 37, 40, 42 or 45°C). The cultures were exposed to a thermal stress of 6 h (a period equivalent to three doubling times of Xcc) under agitation (200 rpm). At the end of the shift, the flasks were returned to the 30°C incubator. OD_600 nm_ measures were taken every 6 h from the start of the thermal stress, until all cultures reached the decline phase (36 h). Viable cells (CFU/mL) were calculated by serial dilution and plating on NYG‐agar. Three independent experiments were conducted for each temperature to be tested. The temperatures inside the incubation chambers were monitored using a mercury thermometer immersed in 100 mL of water contained in a 125 mL Erlenmeyer flask, to mimic the culture flasks.

### Microscopy

Wild‐type Xcc, and the mutants Xcc *amy*::pPM2a‐*zapA* and Xcc *parB*::pAPU3 were cultivated as described in the growth curve section. Six flasks were prepared for each strain to be tested and when the cultures reached the OD_600 nm_ of ~0.4, three flasks were subjected to a 6 h temperature shift at 20°C, 37°C, 40°C, 42°C, or 45°C, while the remaining three flasks were kept at 30°C to serve as internal experimental control. Right at the end of the shifts, three slides were prepared, one for each of the triplicates, for microscope analyses (20 *μ*L of cell culture were dropped onto 1X PBS/1% agarose‐covered slides). Cells were visualized in the magnification of 100× using an Olympus BX‐61 microscope equipped with an orca‐flash2.8 camera (Hamamatsu, Higashi‐ku, Hamamatsu City, Japan). Images were captured and processed using the software CellSens Dimension ver. 11 (Olympus Latin America, INC., Miami, Florida, USA).

### Pathogenicity tests

The plant host used in the pathogenicity tests was the sweet orange Natal [*Citrus sinensis* (L.) Osbeck]. Citrus plants were cultivated under greenhouse conditions at 25–35°C. Xcc cells were cultivated in NYG‐medium until the OD_600 nm_ of ~0.4 (10^8 ^CFU/mL), being subsequently subjected to thermal stress as described in the growth curve section. As soon as the time limit for the thermal stress finished, cell cultures were diluted 1000× in saline and inoculated by infiltration on the abaxial surface of leaves using needleless 1 mL syringes. Symptoms were observed over the course of 3 weeks.

### Data analysis

The average cell length for each thermal stress treatment (20, 37, 40, 42, and 45°C) was calculated by measuring at least 200 cells per treatment. Controls at 30°C were run alongside each treatment to minimize environmental fluctuations and 200 cells were also measured to determine the average cell length for each control. The effect of each thermal stress was determined by comparing the averages of cell length of the treated cells with its internal control (kept at 30°C) applying the *t*‐test of Student (*P *≤* *0.05). Growth curves for Xcc and *t*‐test analyses were conducted using GraphPad‐Prism 6 (La Jolla, CA, USA).

## Results

### Temperature influence over the growth of *Xanthomonas citri* subsp. *citri*

**Figure 1:**
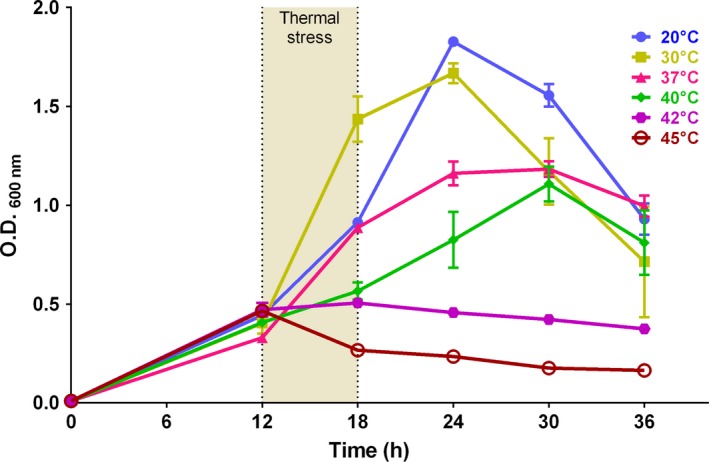
Growth curves of *Xanthomonas citri* subsp. *citri* (Xcc) cultivated at various temperatures. Xcc was cultivated in NYG‐medium at 30°C; upon reaching the OD_600 nm_ of ~0.4 (12 h), cultures were transferred to different temperatures as indicated. Thermal stress was carried out for a period of 6 h (from 12–18 h) with subsequent return to 30°C (from 18–36 h). OD_600 nm_ measures were scored every 6 h. Dots correspond to the averages determined from three independent experiments, while the vertical bars represent the standard deviation.

In order to investigate the effects of temperature shifts on the physiology of Xcc, we subjected the bacterium to different thermal stress during growth (Fig. 1). Prior to thermal stress, the cultures were at the end of the lag phase and at the beginning of the exponential phase as judged by the patterns of the curves around the time 12 h. The growth behavior of Xcc cultivated in optimal laboratory conditions was determined in pilot experiments and is represented by the yellow line. At the beginning of the exponential phase (12 h), cultures were exposed to the different temperatures as illustrated on the left‐hand side of Figure 1. As a result, we observed that thermal stress induced a flattening of the exponential phase (12–18 h; compare the shapes of the curves within the interval of 6 h after the beginning of thermal stress). Note that the higher the temperature, the lower the growth rate of the cultures. Furthermore, we see a clear sign of death of the cultures treated at 45°C, as their OD_600 nm_ values decreased sharply.

Right at the end of the thermal stress treatment, cell samples were collected to check for viability and the ability to resume growth. Primarily, we did not detect any difference in the number of cells determined for the cultures treated at 20 and 37°C when compared with those of the control cultures (Table 1). The cell counting for the three cultures (20, 30, and 37°C) were in the same order of magnitude at the end of the shifts (10^9^ CFU/mL). However, significant differences in cell number were observed for the cultures exposed to 40°C and above. Exposure of Xcc to 40°C led to a drop in the cell counting of one order of magnitude (10^8^ CFU/mL), while incubation at 42°C decreased the count in two orders of magnitude (10^7^ CFU/mL) when compared with the control cell counting. Regarding the highest temperature tested in our experiments, no cells could be rescued on the plate after exposure of cultures to 45°C, corroborating the drop in OD_600 nm_ values observed during the thermal stress.

**Table 1:** Cell viability after the thermal stress

	Viable cells (CFU^a^/mL ± SD^b^)
20°C	2.17 × 10^9^ ± 2.08 × 10^8^
30°C	1.40 × 10^9^ ± 1.00 × 10^8^
37°C	1.00 × 10^9^ ± 1.00 × 10^8^
40°C	4.33 ×10^8^ ± 5.77 × 10^7^
42°C	6.67 × 10^7^ ± 5.70 × 10^6^
45°C	Undetectable

a Colony‐forming unit.

b Standard deviation.

To study the behavior of growth of Xcc after the thermal stresses, cultures were returned to 30°C and monitored for further 18 h (Fig. 1, from 18 h to 36 h). For the cultures treated at 20°C, we noticed only a retardation of growth during the stress period (Fig. 1; compare the blue and yellow lines in the interval between 12–24 h), since these cultures were able to resume growth to similar extents as the control, reaching very close OD_600 nm_ values at 24 h. In both situations (control and 20°C), the cultures displayed a short stationary phase around 24 h, and entered the death phase together at the same time point (24 h). Xcc cells exposed to 37°C were not able to reach the same OD_600 nm_ values as the control (compare the reads at the peak of 24 h), and in addition, they displayed a prolonged stationary phase from 24–30 h. In the cultures submitted at 40°C, the exponential phase was apparently prolonged, with a short stationary phase around 30 h before reaching the decline phase. Finally, at the highest temperatures tested, growth could not be resumed after the stress, and in fact, cultures entered the death phase right at the end of the thermal stress (42°C), or even during the shift (45°C).

### Thermal stress leads to altered cell morphology

**Figure 2:**
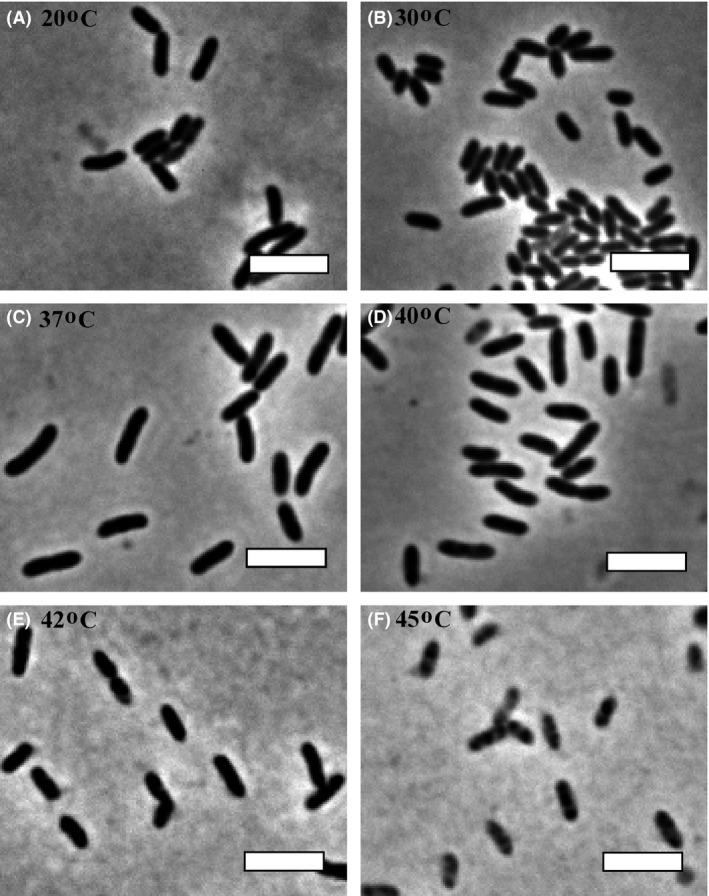
Cell morphology of *Xanthomonas citri* subsp. *citri* (Xcc) cultivated at different temperatures. Xcc was cultivated as described in Figure 1. After the thermal stress, cell samples were analyzed by phase contrast microscopy. Magnification 100×; bar = 4 *μ*m.

Since temperature can induce several physiological alterations in bacteria, we decided to investigate the cell morphology of Xcc following thermal stress (the equivalent of time 18 h in Fig. 1). At first inspection, we did not detect any drastic alteration on cell morphology induced by the thermal treatments (Fig. 2). Nonetheless, we did observe dark clumps (inclusion bodies) within the cells kept at 45°C. Considering that at this temperature Xcc loses viability, the disorganization of cellular contents may reflect the overall protein denaturation. We also noticed that cells cultivated at temperatures differing from 30°C had an apparent increase in size. To confirm this, we compared the averages of cell length obtained for each culture exposed to the different treatments with the average cell size of the control kept at 30°C (Table 2). As a result, all the averages calculated for the treatments differed significantly from their respective controls. Note that the average cell length for the 37 and 40°C treatments was above 2.0 *μ*m, representing an increase in size of more than 33%. The differences in size observed for the 20, 42, and 45°C treatments were less conspicuous if compared to the control, but still, cells were significantly longer.

**Table 2:** Cellular length of *Xanthomonas citri* subsp. *citri* (Xcc) cultivated at different temperatures

		Minimum (*μ*m)	Maximum (*μ*m)	Mean (*μ*m) ± SD
20°C	Treatment	1.02	2.72	1.802 ± 0.285^a^
	Control^b^	0.92	2.08	1.373 ± 0.212
37°C	Treatment	1.34	3.59	2.208 ± 0.411^1^
	Control	0.75	2.22	1.379 ± 0.295
40°C	Treatment	1.16	3.81	2.069 ± 0.441^1^
	Control	1.03	3.05	1.606 ± 0.297
42°C	Treatment	1.14	2.91	1.836 ± 0.354^a^
	Control	1.05	2.99	1.681 ± 0.362
45°C	Treatment	1.09	2.68	1.782 ± 0.328^a^
	Control	0.93	2.38	1.512 ± 0.293

Average cell length for each treatment was acquired measuring at least 200 cells (*n* = 200).

a Averages from the treatments differed significantly from the averages of their respective controls (*t* test of Student; *P* < 0.05).

b Controls were run alongside each treatment at 30°C to minimize environmental fluctuations.

### Essential bacterial processes are perturbed by the thermal stress

Since an increase in cell size may suggest disruption of chromosome segregation and/or cell division (Silva et al. 2013; Ucci et al. 2014), we evaluated the influence of temperature on these processes in Xcc. To analyze the effect of thermal stress on chromosome segregation, we repeated the experiment above, but now using the mutant strain Xcc *parB*::pAPU3 (Ucci et al. 2014). Xcc *parB*::pAPU3 expresses ParB‐GFP, a fusion protein that binds to the replication origin of the bacterial chromosome (centromere) and allows the visualization of the dynamics of chromosome segregation.

**Figure 3:**
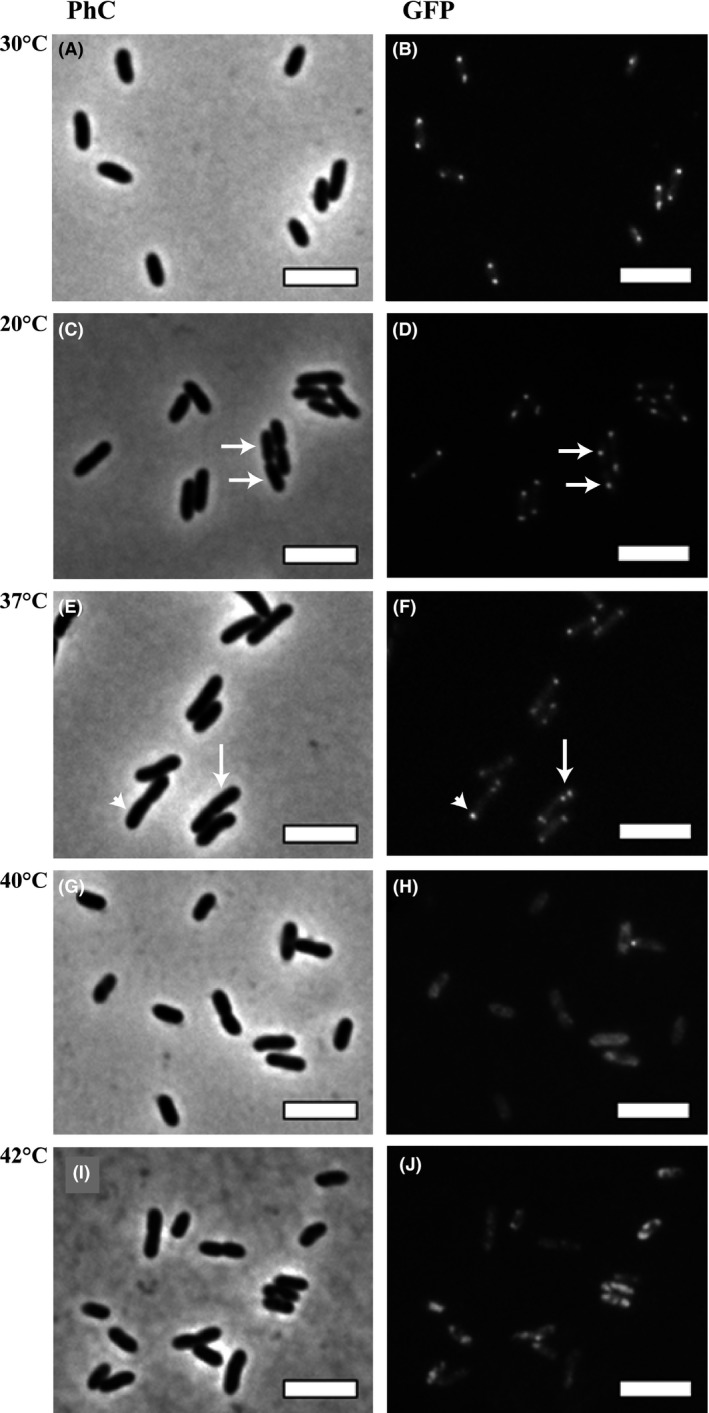
The influence of temperature on the centromere of *Xanthomonas citri* subsp. *citri* (Xcc). The Xcc *parB*::pAPU3 mutant, expressing ParB‐GFP, was cultivated in NYG‐medium at 30°C until the OD_600 nm_ of ~0.4; cultures were transferred to different temperatures as indicated, and thermal stress was carried out for a period of 6 h. Cells were visualized immediately after the shifts. Panels: A–B) 30°C, C–D) 20°C, E–F) 37°C, G–H) 40°C, and I–J) 42°C; (PhC) phase contrast microscopy and (GFP, green fluorescent protein) fluorescence microscopy. Magnification 100×; bar = 4 *μ*m.

Following thermal stress (the equivalent of time 18 h in Fig. 1), the pattern of ParB‐GFP observed for the control (Xcc *parB*::pAPU3 cultivated at 30°C) was as previously reported (Ucci et al. 2014). Here, ParB‐GFP was seen mostly as two bright foci per cellular compartment in practically all the cells (Fig. 3A and B). In these cells, a chromosome replication event is in course and the concomitant segregation of replicated origins toward opposite cellular poles position the ParB‐GFP foci one in each of the poles. A similar phenotype was observed in the cells treated at 20°C, where in general two foci are observed per cellular compartment (Fig. 3C and D). Note for the cells that are about to divide (3C and 3D, white arrows show cells linked with a septum under constriction), the ParB‐GFP foci (e.g. replication origins) are already segregated to the future daughter cells. Noteworthy, the exposure of Xcc *parB*::pAPU3 to 37°C, altered the pattern exhibited by the control in a bacterial sub‐population, in which we now see up to 4 centromeres (ParB‐GFP foci) per cellular compartment (Fig. 3E and F, arrow). The frequency of this cell type was ~5% (*n* = 400), which contrasts with a frequency of less than 1% in cultures kept at 30°C. The presence of three foci per cellular compartment (3E and 3F, arrowhead) was also observed, and it is due to the superimposition of two dots in some pictures. The presence of more than two centromeres per cellular compartment suggests a delay in cellular division leading to the accumulation of replicated origins per cell. Finally, the typical ParB‐GFP pattern could not be detected in cells treated at 40 and 42°C (Fig. 3H and J). Here, only diffuse fluorescence was observed, which indicates that the bacterial centromere has been dissolved.

**Figure 4:**
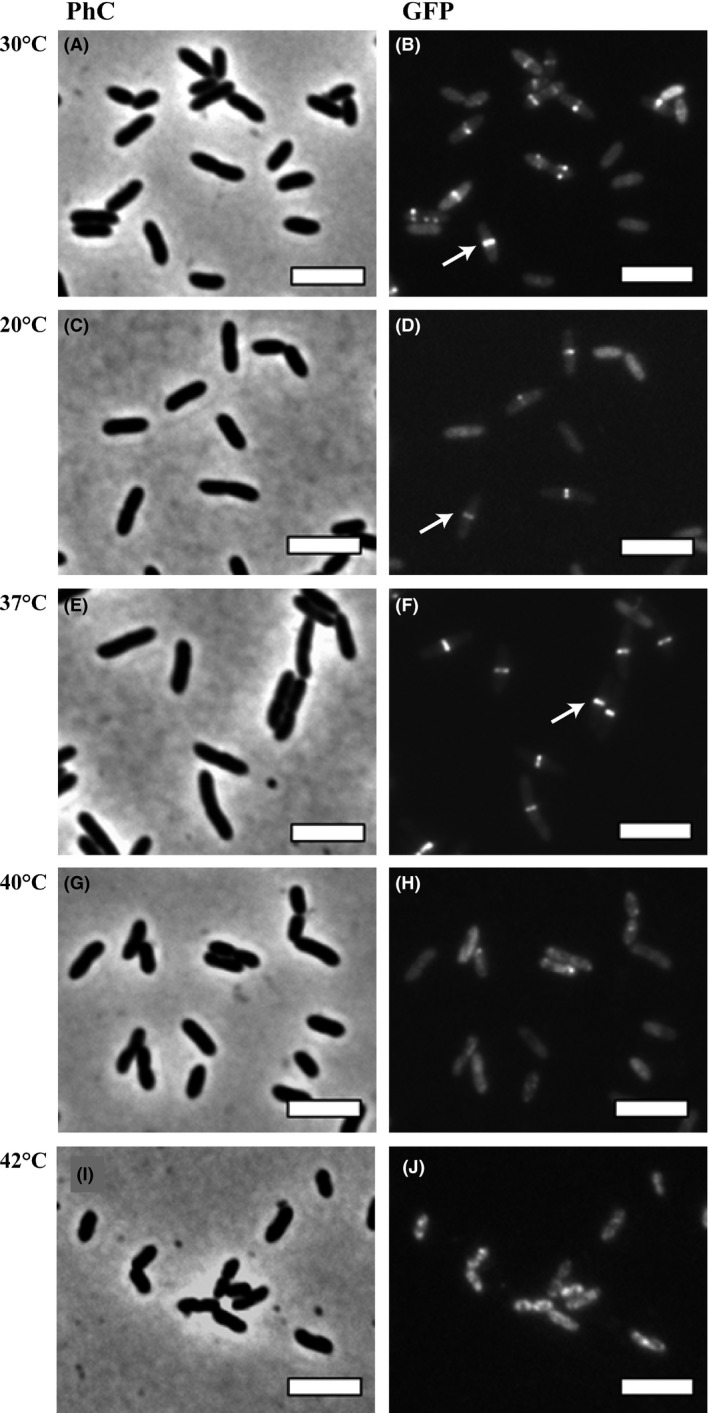
Temperature shifts induce cell division arrest in *Xanthomonas citri* subsp. *citri* (Xcc). The Xcc *amy*::pPM2a‐zapA mutant strain, expressing GFP‐ZapA, was cultivated in NYG‐medium at 30°C until the OD_600 nm_ of ~0.4; cultures were transferred to different temperatures as indicated, and thermal stress was carried out for a period of 6 h. Cells were visualized by (PhC) phase contrast microscopy and (GFP, green fluorescent protein) fluorescence microscopy immediately after the shifts. Panels: A–B) 30°C, C–D) 20°C, E–F) 37°C, G–H) 40°C, and I–J) 42°C. Magnification 100×; bar = 4 *μ*m.

To further characterize if temperature was really delaying cell division, we analyzed a mutant strain of Xcc labeled for the septum in a similar set of experiments. Xcc *amy*::pPM2a‐*zapA* expresses GFP‐ZapA, a protein that associates with the cell division factor FtsZ allowing the visualization of the bacterial septum formed during division (Martins et al. 2010). At first inspection, we did not detect any difference in the pattern of septum placement in cells derived from the treatments at 20, 30°C (control), and the 37°C (Fig. 4B, D, and F, arrows). However, the three cultures differed in the proportion of the cells displaying the divisional septum. For the cultures subjected to 20°C or kept at 30°C, ~30% (*n* = 400) of the cells were dividing (with visible septa), while nearly all the cells had a visible septum after incubation at 37°C (Fig. 4, compare panels 4B, D, and F, and Figure S1). The proportion of cells with septa labeled after the thermal stress at 20°C and in the control reflect nonsynchronized cultures in which the sub‐populations of cells are in fact dividing. Remarkably, thermal stress at 37°C produced an arrest in cell division, which suggests synchronization. Thermal stresses at 40 and 42°C led to a complete loss of the septal structures, where the fluorescence of GFP‐ZapA seemed delocalized within the cells (Fig. 4H and J).

### Thermal stress prevents plant colonization

**Figure 5:**
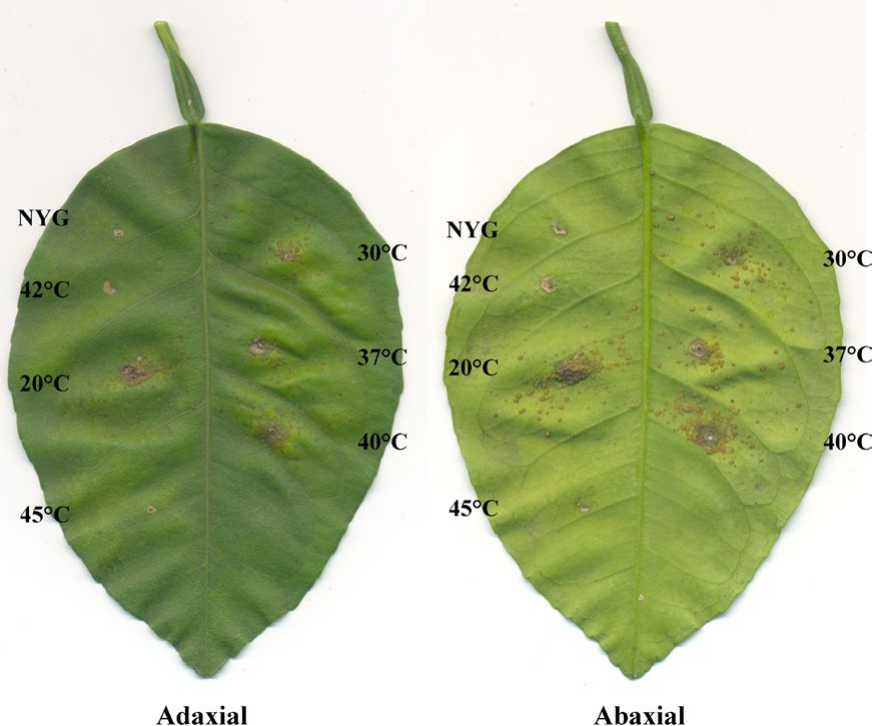
Ability to induce disease in citrus after thermal stress. Wild‐type *Xanthomonas citri* subsp. *citri* (Xcc) was cultivated and subjected to different temperature shifts as described in Figure 1. After the thermal stress, the cell concentration was adjusted to 10^4^ CFU/mL, and Xcc suspensions were infiltrated in leaves of sweet orange Natal; here we show a representative experiment. Analyses were done in triplicate. NYG = NYG‐medium.

Considering that at 40°C there is a decrease in the growth rate of Xcc, and at this temperature the bacterium cannot divide, we wanted to evaluate the influence of the thermal stress on the pathogenicity/virulence of this plant pathogen. To investigate this, wild‐type Xcc was subjected to different temperatures as described earlier, and right after the stress period (the equivalent of time 18 h in Fig. 1), cells were infiltrated into the leaves of a susceptible host (Fig. 5). As a result, cells exposed to 20 and 37°C were equally competent to induce the symptoms of citrus canker as the control grown at 30°C. Cells derived from these cultures induced the typical brownish corky‐like lesions on both leaf surfaces (compare the absence of symptoms in the area infiltrated with the culture medium NYG). Surprisingly, the treatment at 40°C did not eliminate the ability of Xcc to colonize the host, since cells subjected to this temperature produced as much symptoms as the cells kept at 30°C or exposed to 20 or 37°C. Finally, we did not detect any symptoms in the regions inoculated with cells derived from the cultures treated at 42 and 45°C.

## Discussion

Considering that Xcc spends part of its life cycle as an epiphyte, bacterium has to deal with environmental temperature oscillations that are quite different from the optimum cultivation temperature used in the laboratory (28–30°C). By investigating the effects of thermal stress for periods of 6 h on the growth of Xcc, we observed: first, the cell cycle is arrested at division when the bacterium is exposed to 37°C, and in addition, the cell division arrest detected does not seem to interfere with the ability of Xcc to colonize the host citrus. Secondly, cell viability is compromised at temperatures above 40°C, and Xcc loses the ability to produce the disease in citrus when exposed to 42°C.

Our studies demonstrate that an increase of 7°C from the optimal cultivation temperature led to a cell cycle synchronization in Xcc without interfering with the virulence of this plant pathogen. Synchronization has important meanings, since it allows for a more comprehensive analysis of cellular events that take place during defined periods of the cell cycle (Helmstetter et al. 1992; Withers and Bernander 1998; Ferullo et al. 2009; Lin et al. 2012; Schrader and Shapiro 2015). By using fluorescent cell division and chromosome segregation markers (Martins et al. 2010; Ucci et al. 2014), we showed that at 37°C, Xcc exhibits a cell cycle halt at the predivisional stage, since the majority of the cells had septa labeled. This cell cycle arrest induced by thermal stress may have also interfered with the DNA replication and/or the chromosome segregation processes, since Xcc cells displayed an increased number of sister origins side‐by‐side within rods (Fig. 3E and F, white arrow; compare with dividing rods in 3C and D, white arrows). According to our previous report (Ucci et al. 2014), Xcc has an asymmetric mode of chromosome replication, where two origins side‐by‐side indicate initiation of DNA replication. Arrest of DNA replication at initiation has been reported in *E. coli* either using thermal stress with *dnaC* mutants or by inducing the stringent response in wild‐type cells (Withers and Bernander 1998; Ferullo and Lovett 2008; Ferullo et al. 2009). On the contrary, the thermal stress in our experiments was conducted with cells carrying normal alleles coding for components of the replisome, and the accumulation of sister origins observed in Xcc exposed to 37°C indeed suggests the perturbation of either the replisome or the segrosome functions. Finally, our results contrast sharply with the observation that in wild‐type *E. coli*, upshifts of 10 degrees or more from the optimum cultivation temperature apparently stimulates initiation of chromosome replication (Gonzalez‐Soltero et al. 2006). The reason for that still needs to be investigated.

The fact that thermal stress at 42°C compromised the ability of Xcc to colonize the host citrus may constitute an alternative method for eliminating this plant pathogen from plant material. Besides the high cost and effectiveness of the strategies to control citrus canker in the field (Ferreira and Belasque 2011; Behlau et al. 2014), another question of concern has been the transit of plant aerial parts as ornamentals and fruits among areas, and the risk of introducing Xcc into regions free of the pathogen (Gottwald et al. 2002a,b, 2009). Anco et al. (2014) reported a comprehensive analysis about the effects of thermal stress, allied to the presence of the disinfectant Pro‐San, on the viability of Xcc. Research on this field is increasing, since post‐harvest treatments may eliminate sources of bacterial inoculum from plant material to a minimal necessary to make transit of plant parts possible. Thermotherapy (40–42°C/~10 days) was recently used to eliminate ‘*Candidatus* Liberibacter asiaticus’ (*Ca*. L. asiaticus) from citrus, a technique with great potential to disinfect plant material (Hoffman et al. 2013). Our data showed that a similar temperature range (40–42°C) promoted a reduction of Xcc cell viability, and impaired its ability to colonize the host. One explanation for this phenomenon is the disassembly of protein complexes engaged in vital cellular processes such as cell division and chromosome segregation (Fig. 3 and 4, panels H and J). In addition to this, other cellular complexes may be suffering the same effect as well.

The present work described a strategy that constitutes a simple method for cell synchronization of Xcc. Moreover, it may be explored as an alternative and easy method to clean plant material. Using temperature shifts, an optical microscope prepared for fluorescence studies, and Xcc strains labeled for the septum and replication origins (Martins et al. 2010; Ucci et al. 2014), one can devise experiments to explore distinct aspects and moments of the cell cycle. Here we showed data derived from thermal stresses conducted over periods of 6 h. However, the duration of the stress can be calibrated using microscope inspections in order to rescue cells with specific morphologies and/or physiological properties.

## Conflict of Interest

None declared.

## Supporting information


**Figure S1.** Thermal stress induces cell division arrest in Xcc.
