# Supplementary figures and images for: Temperature stress promotes cell division arrest in Xanthomonas citri subsp. citri
Source: Microbiologyopen. 2015 Dec 13;5(2):244–53. doi: 10.1002/mbo3.323 (PMC4831469; doi:10.1002/mbo3.323)

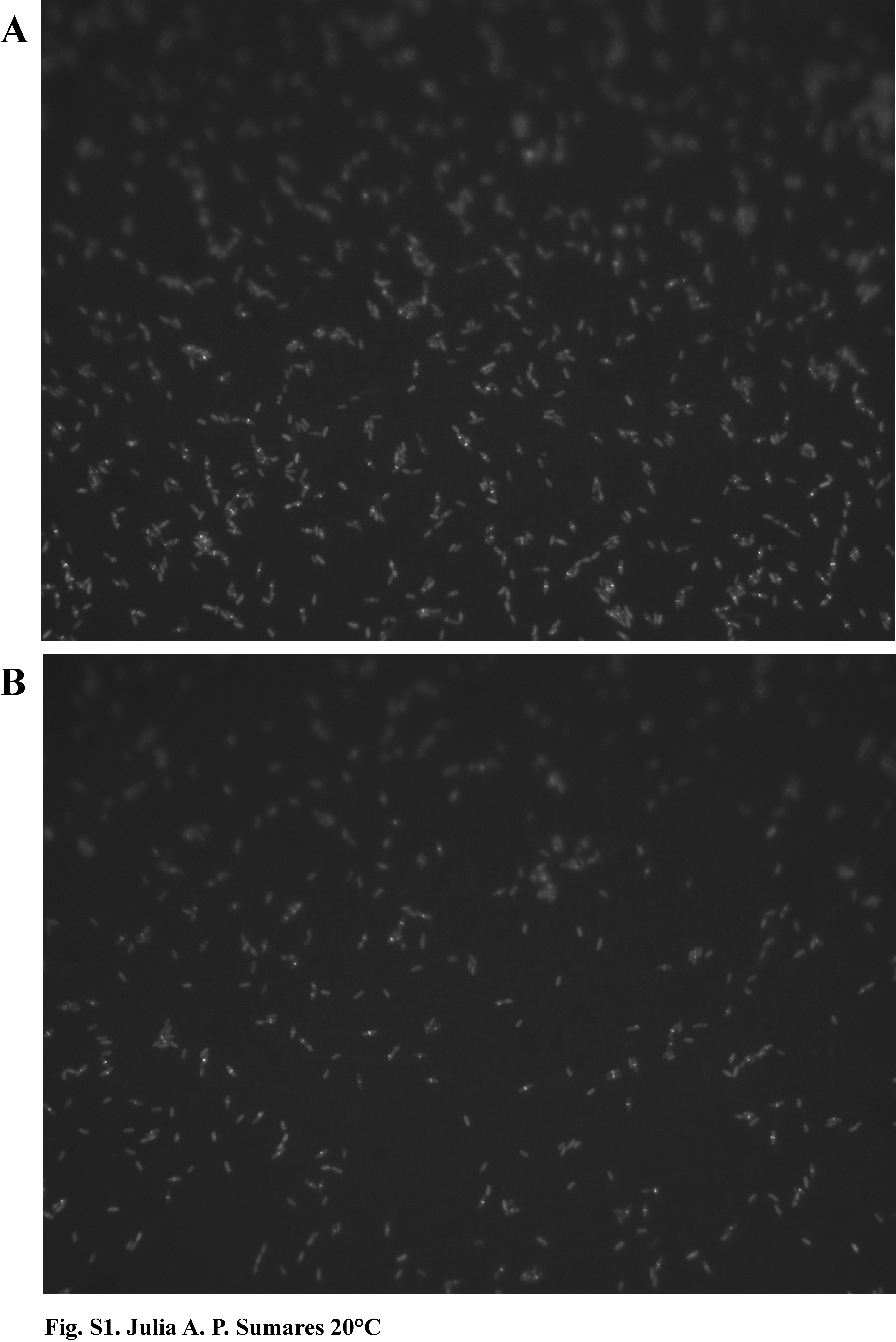

Supplement: Supplementary file 1 — Figure S1. Thermal stress induces cell division arrest in Xcc. [file MBO3-5-244-s001.tif]

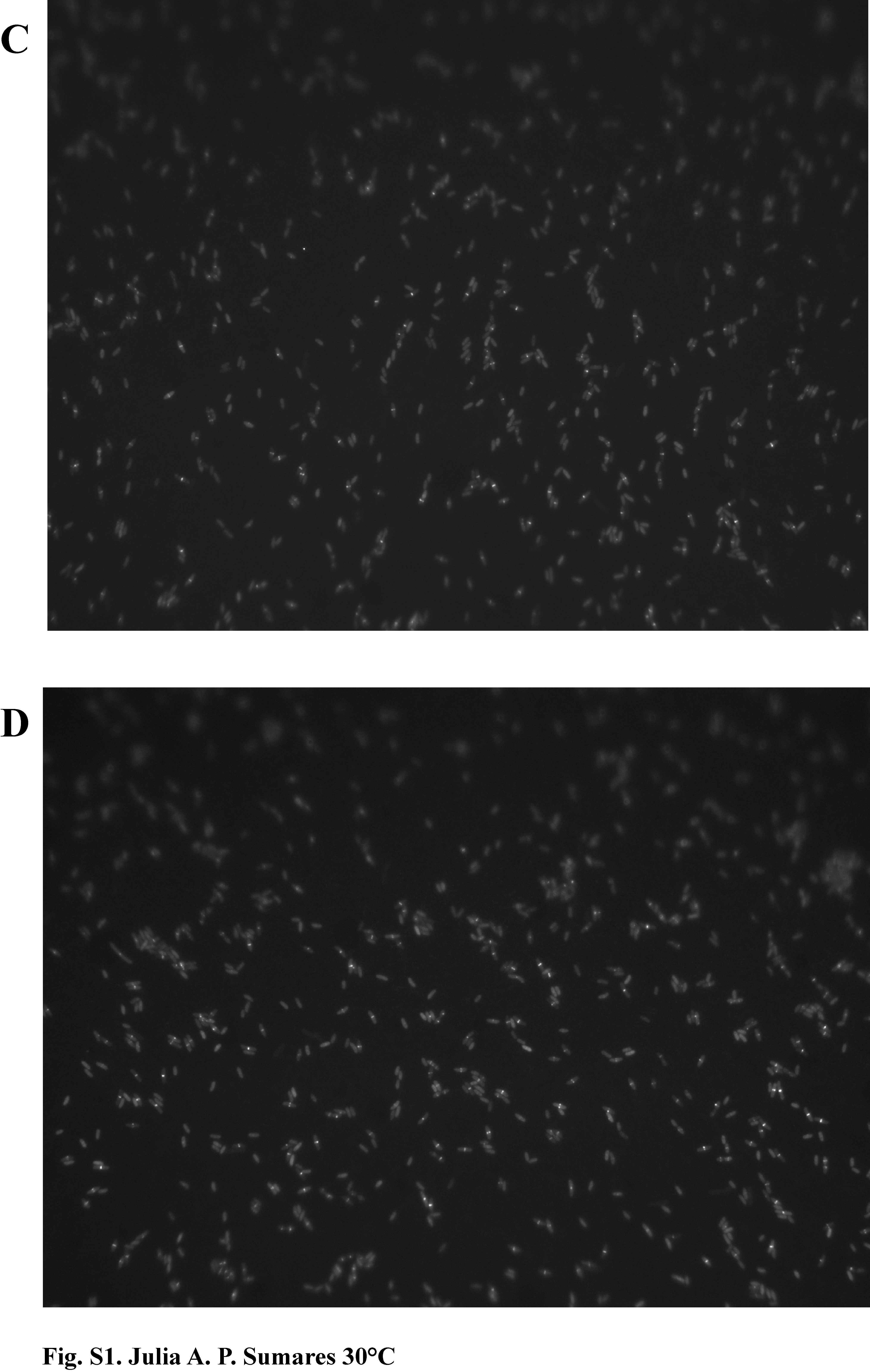

Supplement: Supplementary file 2 [file MBO3-5-244-s002.tif]

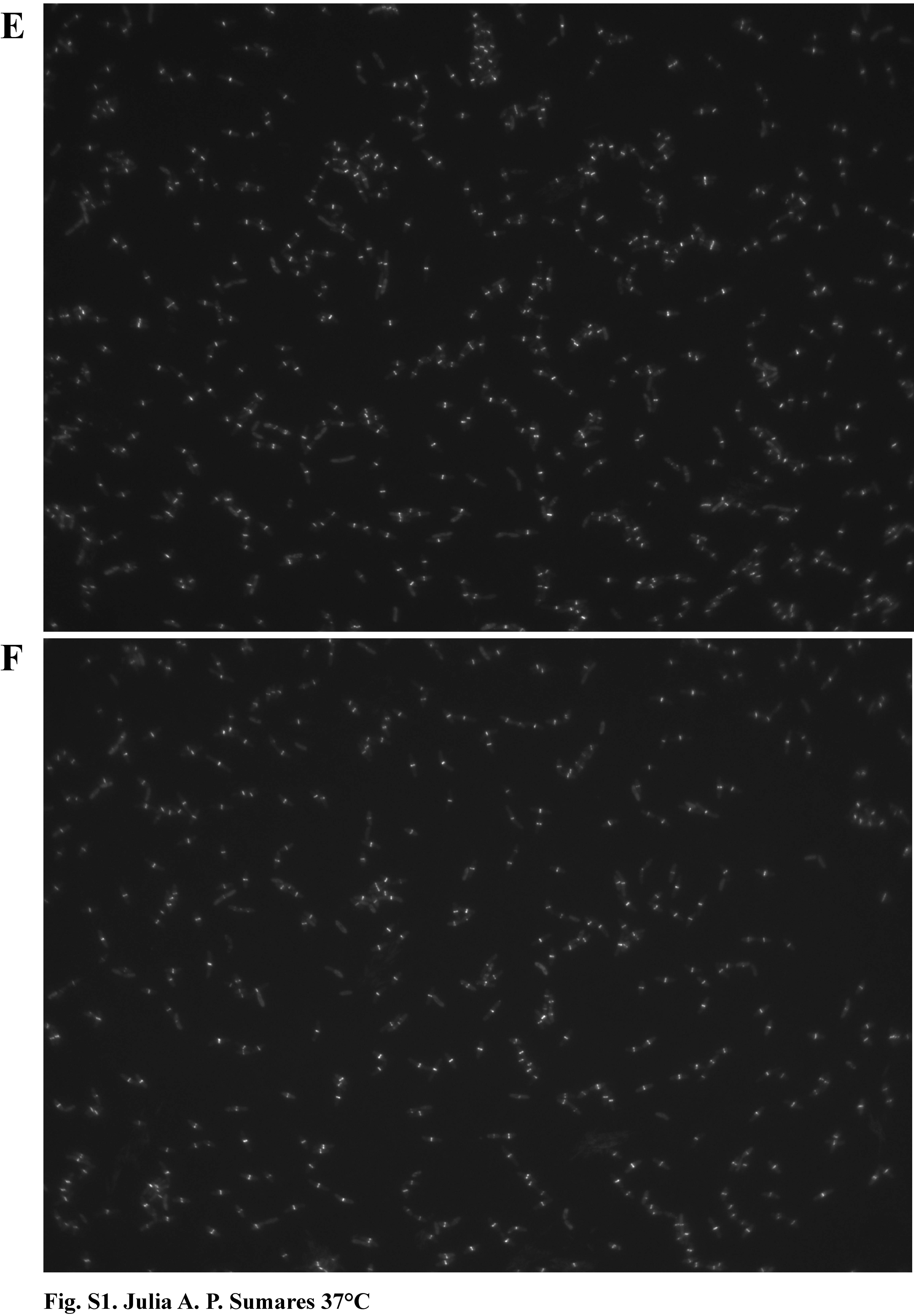

Supplement: Supplementary file 3 [file MBO3-5-244-s003.tif]

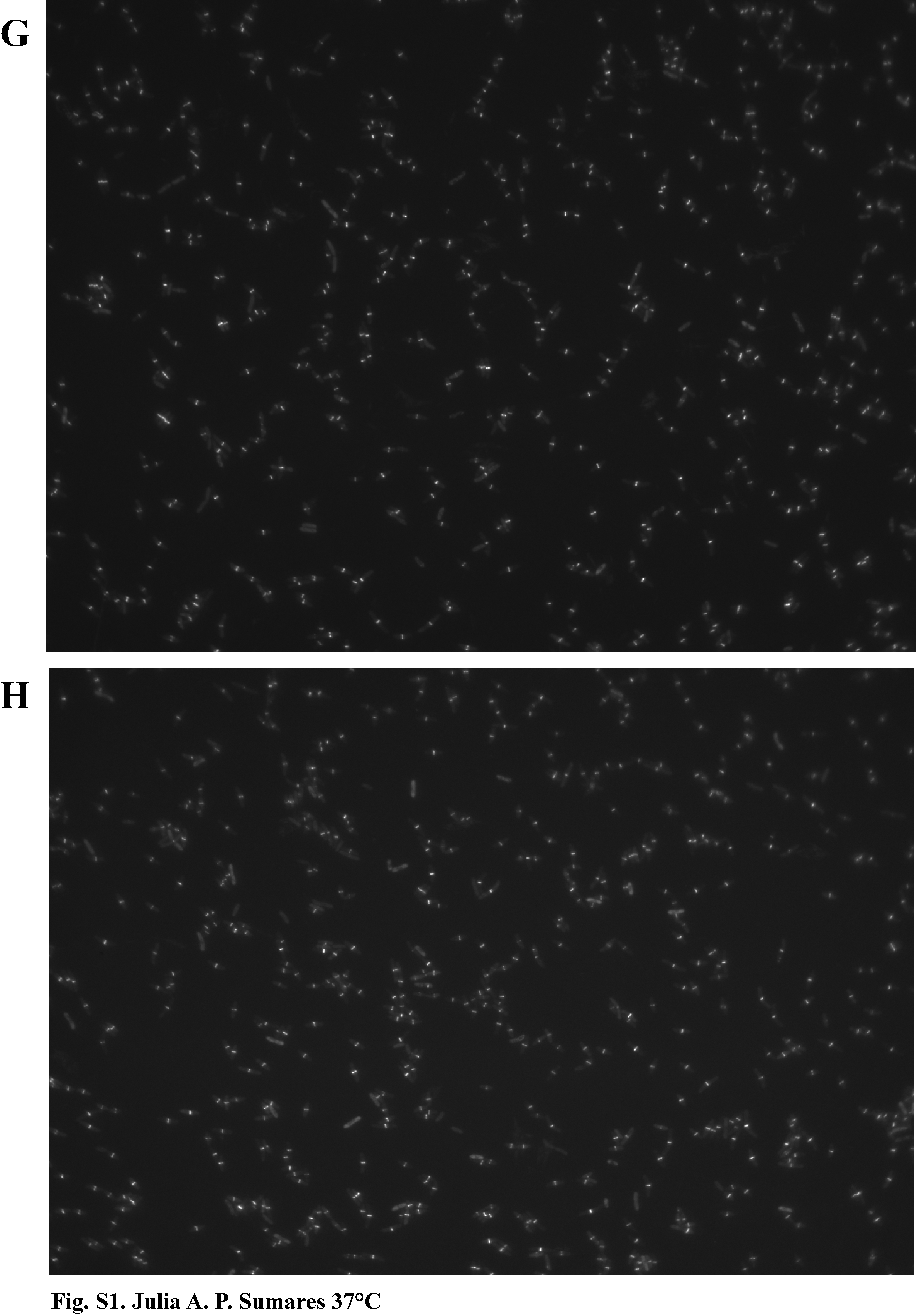

Supplement: Supplementary file 4 [file MBO3-5-244-s004.tif]

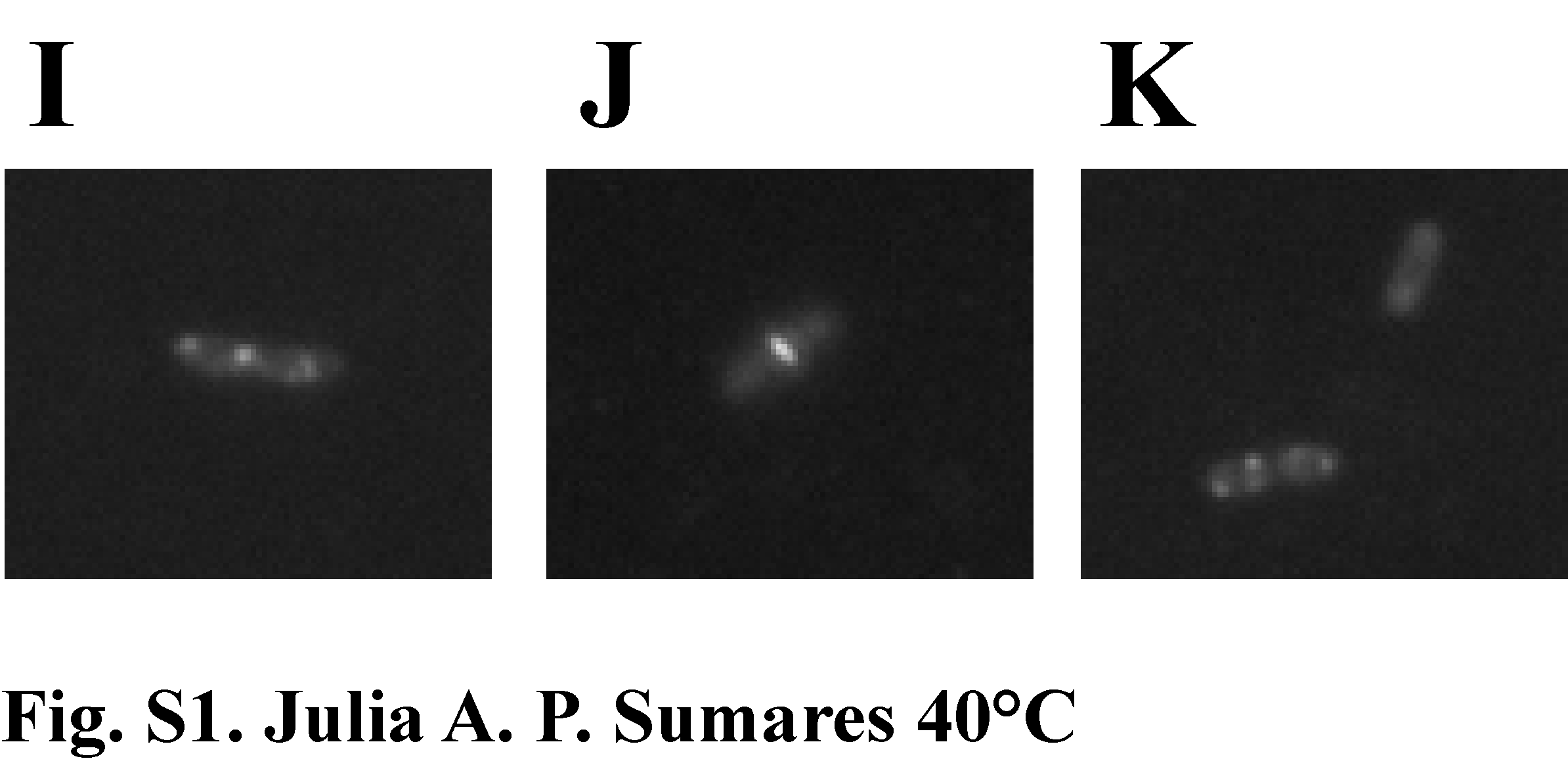

Supplement: Supplementary file 5 [file MBO3-5-244-s005.tif]
